# Paramylon and Other Bioactive Molecules in Micro and Macroalgae

**DOI:** 10.3390/ijms23158301

**Published:** 2022-07-27

**Authors:** Laura Barsanti, Lorenzo Birindelli, Paolo Gualtieri

**Affiliations:** Istituto di Biofisica, Area della Ricerca, Consiglio Nazionale delle Ricerche, Via Moruzzi 1, 56124 Pisa, Italy; laura.barsanti@ibf.cnr.it (L.B.); lorenzo.birindelli@ibf.cnr.it (L.B.)

**Keywords:** algae, pigments, PUFAs, polyphenols, polysaccharides, vitamins, *Euglena gracilis*

## Abstract

Many algae synthesize compounds that have exceptional properties of nutraceutical, pharmacological, and biomedical interest. Pigments, fatty acids, phenols, and polysaccharides are among the main compounds investigated so far. Polysaccharides are the most exploited compounds, widely used in pharmaceutical, food, and chemical industries, which are at present entering into more advanced applications by gaining importance, from a therapeutic point of view, as antioxidant, antimicrobial, antitumor, and immunomodulatory agents. Establishing algae as an alternative supplement would complement the sustainable and environmental requirements in the framework of human health and well-being. This review focuses on the proprieties and uses of the main micro- and macroalgae metabolites, describing their potential for application in the different industrial sectors, from food/feed to chemical and pharmacological. Further, current technologies involved in bioactive molecule extraction strategies are documented.

## 1. Introduction

Algae can be simply grouped into two types based on cellularity: microalgae, which includes unicellular prokaryotic and eukaryotic species, and macroalgae, which includes only eukaryotic multicellular species. All of these organisms can be defined as light-driven cell factories that synthesize bioactive compounds consisting of primary metabolites, i.e., molecules that play a direct role in the algae metabolism (lipids, amino acids, and carbohydrates) and specialized secondary metabolites (pigments and vitamins) [1]. Humans are unable to synthetize most of these molecules or synthetize them in too low amounts, and external administration in the diet is strongly recommended to avoid dangerous deficits. Hence, the importance of safe dietary supplements with long-term physiological health benefits, even though their effects can be mild compared to those of pharmaceutical products [2,3,4].

Humans have utilized algae for hundreds of years as food, fodder, remedies, and fertilizers, as evidenced by macroalgae fossil remains recovered in a late Pleistocene settlement in southern Chile, which date back to 13,000 years ago. Other archeological data confirm the consumption of cyanobacteria (*Arthrospira*) in Mexico by Aztecs in the fourteenth century and Chad by the Kanembu people in the ninth century [1]. In Japan, about 50 species of algae are used daily as foods, with an estimated annual consumption per person up to 2 kg. This ancient tradition that dates back to the IV century allowed a large number of epidemiological surveys showing the health benefits of algae consumption and the absence of long-term side effects [1]. According to the results of these studies, Japanese people have one of the lowest rates of obesity in the world, a very long life expectancy (>80 years), low rate of cardiovascular diseases, and a very low rate of several types of cancer [1]. All this evidence validates the importance of algae as edible resources with important medicinal properties since the beginning of human history and supports their role as sustainable food and bioactive ingredients.

Nowadays, the global production of algae is dominated by marine macroalgae grown in both marine and brackish waters, and cultivation has overshadowed the production of algae collected from the wild, which accounted for only 3% of total production in 2019 [5,6]. A few species belonging to eight genera of Rhodophyta and Ochrophyta dominate the world macroalgae production: *Eucheuma* spp., *Kappaphycus alvarezii*, *Gracilaria* spp., *Porphyra* spp., *Pyropia* spp., *Saccharina japonica syn. Laminaria japonica*, *Undaria pinnatifida*, and *Sargassum fusiforme*. A small production of Chlorophyta is due primarily to five genera: *Caulerpa*, *Monostroma*, *Enteromorpha*, *Capsosiphon,* and *Codium*. As to microalgae, large-scale commercial production of biomass is limited to *Arthrospira* (Cyanobacteria), *Dunaliella*, *Haematococcus,* and *Chlorella* (Chlorophyta), which are cultivated in open ponds and/or closed systems at farms located around the world. Nowadays, the algal global world market value is about USD 6 billion [7,8,9].

All of these cultivated algae, as a whole, possess a high-quality nutritional value; hence, they can be considered functional food, i.e., food that beneficially affects one or more target functions in the body. Functional foods provide dietary benefits beyond their macronutrient content and have been recognized to promote excellent health, decrease the risk of mainly non-communicable diseases, such as cardiovascular diseases, cancers, and diabetes, and enhance cost-effective care by promoting a better quality of life [10,11]. Moreover, because of their content in bioactive molecules, such as ω3 essential polyunsaturated fatty acids (PUFAs) (e.g., docosahexaenoic acid (DHA) and eicosapentaenoic acid (EPA)), sterols, minerals (e.g., Na, K, Ca, Mg, I), carotenoids (e.g., β-carotene, astaxanthin), essential vitamins (e.g., A, B_1_, B_2_, C, D, E), polyphenols, polysaccharides (e.g., alginate, fucoidan, β-glucan), algae can also be considered a nutraceutical (a crasis of the terms “nutrition” and “pharmaceutical”). Nutraceuticals are products for oral administration, presented in non-conventional food formats, which supplement the diet and provide health benefits in such a quantity that exceeds those that could be obtained from plain foods [12,13].

Many of the bioactive molecules of algae have been tested in clinical trials or are under validation to assess their efficacy in tackling vitality issues, especially in the field of lipid metabolism, oxidative cellular stress, cancer, and neurological and cardiovascular diseases [14,15,16]. Some of the general effects of algal PUFAs include anti-arteriosclerosis, anti-hypertension, anti-inflammation, and immunoregulation; polyphenols have shown bioactivity effects on cancer as antioxidants and anti-inflammatories. Carotenoids also possess similar antioxidant, anti-cancer, and anti-diabetes activities. Among the most important bioactive molecules, β-glucans deserve a special mention because they are considered a very promising alternative to the mainstream use of immunosuppressive drugs for inflammatory diseases. These polysaccharides, in fact, have a potent immunomodulating activity since they can enhance defense mechanisms against infection and simultaneously down-regulate inflammation [17,18].

There is a strong body of science underpinning the health benefits of algae, and new, different compounds synthesized by these organisms are continuously investigated and tested for their applicability in improving health and reducing the risk through prevention. Still, knowledge should be improved, optimizing strain selection through genetic and metabolic engineering for the highest accumulation of bioactive products; moreover, the biological availability of high functional foods and supplements should be assessed for a correct evaluation of how much of the ingested compound is really absorbed, metabolized, and utilized and can exert its biological function [1,19].

In the following, the current technologies involved in bioactive molecule extraction strategies are documented, together with new possible applications. This article is intended as an introduction to the special issue of the International Journal of Molecular Sciences entitled “Glucans, Paramylon and other Algae Bioactive Molecules”.

## 2. Bioactive Compounds

The general beneficial effect of algae is attributed to their content in pigments, PUFAs, polyphenols, polysaccharides, and vitamins (Table 1). This review will be mainly focused on carotenoids (pigments) and PUFAs, which exhibit antioxidant and anti-inflammatory activities, and β-glucans (polysaccharides), which act as immunomodulators.

### 2.1. Pigments

Pigments present in algae can be grouped into three types: chlorophylls, phycobilins, and carotenoids.

Chlorophylls (*a*,*b*,*c*,*d*,*f*) are the essential pigments of photosynthesis, present in light-harvesting complexes of all oxygenic photosynthetic organisms. These molecules consist of a hydrophilic porphyrin head formed by four linked pyrrole rings with a Mg^2+^ atom chelated at the center and a hydrophobic phytol tail. All the chlorophylls harvest light in the blue and red regions of the visible solar spectrum and transduce it into chemical energy. For the molecular structures of the different chlorophylls and their absorption spectra, refer to [1]. Chlorophylls gained popularity as dyeing agents; at the European level, these pigments have a wholesale price averaging EUR 15,000 t^−1^ [7].

Phycobilins possess four pyrrolic rings linearly arranged, and, unlike the chlorophylls, they are strongly covalently bound to a protein. These polar proteins, named phycobiliprotein, possess more than one subunit and more than one phycobilin as a chromophore. They are arranged in phycobilisomes, regulatory protein complexes that harvest light energy and transfer it to chlorophyll *a*. Phycobiliproteins are divided into four main groups depending on the absorption range and type of chromophore: phycoerythrins (λ_max_ 540–570 nm), phycocyanins (λ_max_ 610–620 nm), phycoerythrocyanins (λ_max_ 560–600 nm), and allophycocyanins (λ_max_ 650–655 nm) [1]. Phycobiliproteins are present in many microalgae, especially cyanobacteria and macroalgae (particularly rhodophytes), and have substantial potential as biologically active agents with anti-inflammatory properties, and immunomodulating, anti-cancer, antihyperlipidemic, antioxidative, and angiotensin-converting-enzyme (ACE) inhibitory effects. In the aquaculture industry, phycocyanin has been utilized as a feed supplement in shrimp, fish, and ornamental fish as it contains high nutrients and enhances skin color. Phycobiliproteins are natural additives in the food industry and essential fluorescent probes in biotechnology. They are commercially extracted mainly from the cyanobacterium *Arthrospira* sp. (phycocyanins) and from rhodophytes such as *Porphyridium cruentum*, *Pyropia tenera*, and *Porphyra* sp. (phycoerythrins, phycoerythrocyanins), by means of aqueous, acidic, and alkaline methods, followed by several rounds of centrifugation and recovery using techniques such as ultrafiltration, precipitation, chromatography, and water extraction methods [22,23,24]. Resistant cell walls and the presence of high viscosity and anionic carbohydrates in these algae can substantially hinder the success of the extraction procedure; hence, cell disruption methods and chemical reagents (mechanical grinding, osmotic shock, ultrasonic treatment, and enzyme-assisted hydrolysis) are used in order to improve the efficiency of phycobiliproteins extraction. The overall market of phycobiliproteins for 2018 was USD 112.3 million, and it is expected to double by 2028 thanks to their high commercial value as natural colorants in the nutraceutical, cosmetic, and pharmaceutical industries, and in clinical research, molecular biology, and as natural dyes in the textile industries [7].

Carotenoids are tetra-terpenoid hydrophobic pigments that generally have a C_40_ backbone structure of isoprene units. These molecules can be divided into carotenes (pure hydrocarbons) and xanthophylls (oxygenated carotenes). Examples are carotenes, lycopenes, fucoxanthin and its derivatives (the most abundant carotenoids in the world’s ocean), astaxanthin, zeaxanthin, neoxanthin, lutein, and violaxanthin. For the molecular structures of the different carotenoids and their absorption spectra, refer to [1]. Carotenoids are present in light-harvesting complexes of all algae and serve a key role in their metabolism, protecting the chlorophyll environment from photodamage by quenching free radicals and so inhibiting oxidative injury to cells, tissues, and membranes [1].

There has been much interest in the role of carotenoids in human and animal health, especially for their antioxidant activity in attenuating oxidative damage and preventing damage to lipid bilayers. Fucoxanthin, a carotenoid commonly distributed in brown algae, such as *Undaria pinnatifida*, *Scytosiphon lomentaria*, *Petalonia binghamiae*, and *Laminaria religiosa*, and microalgae, such as *phaeodactylum tricornutum,* is a potent drug candidate and can be utilized as an excellent supplement (e.g., astaxanthin) since it acts as an antioxidant and inhibits neuroblastoma and colon cancer cells [25]. Other macroalgae contain siphonoxanthin (*Codium fragile*), lutein (*Porphyra tenera*), and zeaxanthin (*Ascophyllum nodosum*) [16].

Carotenoids are also the major dietary source of pro-vitamin A, essential for cell growth, embryonic development, vision, and immune system functioning. They have a global market value of about USD 2 billion [7].

A major bottleneck in the exploitation of microalgal biomass is the low productivity of the culture, both in terms of biomass and product concentration. One fundamental reason for this is the slow cell growth rates owing to the inefficient use of strong light. Furthermore, carotenoids are secondary metabolites produced when growth is limited or under stress conditions. A possible solution to this bottleneck is to milk the secondary metabolites from the microalgae, i.e., continuously removing the products from the cells, thereby enabling the biomass to further synthesize them. The milking method developed for *Dunaliella salina* β-carotene uses a two-phase bioreactor [26]. Cells are first grown under normal growth conditions and then stressed by excess light to produce larger amounts of β-carotene. At this stage, the biocompatible organic phase is added, and the β-carotene is extracted selectively via continuous re-circulation of a biocompatible organic solvent (lipophilic compound) through the aqueous phase containing the cells. Because the cells continue to produce β-carotene, the extracted product is continuously replaced by newly synthesized molecules. In contrast to existing commercial processes, this method does not require harvesting, concentrating, and disrupting the cells for extraction of the desired product. Furthermore, the purification of the product is simple, owing to the selectivity of the extraction process. The general application of this process would facilitate the commercialization of microalgal biotechnology and the development of microalgal products.

The properties of the cell membrane play an important role in the contact between biocompatible lipophilic solvents and hydrophobic parts of the cell membrane since this contact might be prevented by the presence of a cell wall and/or hydrophilic parts of the outer membrane. Physiological properties of the cells, such as their capacity for continuous endo- and exocytosis, might also play a role in the milking process. Other considerations are the location and way in which the product accumulates inside the cells and its function in the cell metabolism. A product like chlorophyll would be difficult to extract owing to its location within the thylakoid membranes and because it is strongly bound to other cell components. The extraction of a compound with a protective role for the cells (e.g., β-carotene) will enhance its synthesis. As an example, astaxanthin, well known for its use in aquaculture (e.g., to give salmon flesh a pink color), has nutraceutical importance related to free-radical scavenging, immunomodulation, and cancer prevention [27]. *Haematococcus pluvialis* can produce and accumulate astaxanthin up to 8% of the dry weight (dw) [27]. This concentration within the cell would make the milking of *H. pluvialis* more successful compared with *D. salina*. Still, supercritical fluid extraction with CO_2_ is now used to extract this xanthophyll from *Haematococcus* biomass. Moreover, the cultivation of *H. pluvialis* is more complex and less productive than *D. salina*, involves a two-step process, and the problems associated with the production (expensive biorefinery process, costly production, space, and time consumption) need to be tackled for an efficient scaling up. It goes without saying that productivity, duration of the growth period, biomass harvesting, extraction, purification, and concentration, are a heavy burden on the final cost of the products, and for a sustainable production process, the “high-value product first” approach should be applied, combing the production of more products and using the residual biomass.

### 2.2. PUFAs

Fatty acids, a major component of lipids, are carboxylic acids consisting of a hydrocarbon chain and a terminal carboxyl group. The chain can be either saturated (without double bonds between two carbon atoms) or unsaturated (with double bonds between two carbon atoms). Most naturally occurring fatty acids possess an unbranched chain of an even number of carbon atoms, from 4 to 28. Fatty acids are chemically divided into three main classes of esters: triglycerides, phospholipids, and cholesteryl esters. PUFAs are gaining increasing importance as valuable pharmaceutical products and food supplements thanks to their beneficial effect on human health [13,28]. DHA (22:6 ω3), EPA (20:5 ω3), along with their precursor α-linolenic acid (ALA, 18:3 ω3), are important in the development and functioning of brain, retina, and reproductive tissues in both adults and infants [29]. They can also be used in the treatment of various diseases and disorders, including cardiovascular and inflammatory diseases, as well as cancer. At present, PUFAs are produced commercially from fish oil, but this is an insufficient source of these products, and microalgae provide an optimal alternative source.

Thanks to the presence of specific desaturase enzymes, algae are essentially the only organisms able to produce long chain PUFAs, from 14 to 24 carbons. Humans and other animals cannot convert ALA to EPA and DHA at required levels, so dietary additions of these essential fatty acids are critically important for their health. Seaweeds and microalgal-derived PUFAs, such as ARA (arachidonic acid) and DHA, are added as fortifications to infant formulae. The selling price for PUFAs sold as micronutrients, and anti-inflammatory compounds can be higher than USD 5 million t^−1^ [7,30,31].

Microalgae are the primary producers of EPA and DHA that are eventually accumulated through the various trophic levels. Changes in microalgal lipid content are carried up the food chain, impacting the growth and dietary make-up of zooplankton, crustacean larvae, mollusks, and some fish. This subsequently affects the accumulation of EPA and DHA fatty acids in higher organisms and humans. Consequently, lipid profiles in microalgae play a vital role in maintaining the integrity of the world’s aquatic food webs.

Several heterotrophic microalgae have been used as biofactories for omega-3 fatty acids commercially, but a strong interest also in autotrophic microalgae has emerged in recent years. To date, the ω-3 fatty acid content of numerous microalgae strains has been studied. *Phaeodactylum tricornutum* and *Nannochloropsis* sp. demonstrated an EPA content of up to 39% of total fatty acids. High biomass and commercially acceptable EPA and DHA productivities are achieved with microalgae grown in media with optimized carbon and nitrogen concentrations and controlled pH and temperature conditions. High oil production can be obtained as a result of the high growth rate by controlling nutrients such as glucose, nitrogen, sodium, and other environmental factors, such as oxygen concentration, temperature, and pH, achieving high cell densities and DHA productivities [1].

The heterotrophic marine dinoflagellate *Crypthecodinium cohnii* has a lipid content greater than 20% dw and is known for its ability to accumulate fatty acids that have a high fraction (30–50%) of DHA. Lipids are important components of algal cell membranes but also accumulate in globules in other parts of the cells. Microalga growth and fatty acid formation are affected by medium composition and environmental conditions (e.g., carbon sources). Lipid production occurs under growth-limiting conditions; during linear growth, the cells are stressed owing to nutrient limitations and, therefore, produce more lipids. Furthermore, the lipid quality (DHA concentration) is negatively affected by increases in lipid concentration. The highest quality lipid is obtained when glucose is used as the carbon source and when the cell concentration and lipid content of the cells are the lowest [1].

Milking can also be used for DHA recovery from *C. cohnii* cells. In this process, cells are first grown under the best conditions for growth to achieve high biomass and then stressed to produce higher concentrations of DHA. A biocompatible organic solvent is added during the DHA production stage to extract the product [1].

An EPA production potential has also been found in the genus *Nitzschia* (especially *N. alba* and *N. laevis*). It was reported that the oil content of *N. alba* was as high as 50% of cell dw, and the EPA comprised 4–5% of the oil*. N. laevis* could utilize glucose or glutamate as a single substrate for heterotrophic growth, and the cellular EPA content of the alga in heterotrophic conditions was also higher than that in photoautotrophic conditions, suggesting that this diatom is a good heterotrophic EPA producer [1].

From a production point of view, an increase in microalgal lipid content can be induced by a sudden change in growth conditions. The accumulation of starch and/or lipids reserves is considered a survival mechanism in response to growth-limiting stresses, such as UV radiation, temperature, and shock or nutrient deprivation, as long as light conditions are present that allow efficient photosynthesis. For example, during nutritional deprivation (e.g., nitrogen) and under the provision of light, cellular division of many marine or brackish microalgae is put on hold, and cells begin to accumulate lipids, leading to a 2–3-fold increase in lipid content. Both total lipid and omega-3 fatty acid production can be adjusted by varying growth conditions. The diatom *Phaeodactylum tricornutum* can be induced to increase its lipid level from 81.2 mg g^−1^ of culture dw to 168.5 mg g^−1^ dw, *Nannochloropsis* sp. and *Dunaliella* sp. can achieve a total lipid content of up to 47% and 60% of dw by modifying light intensity, temperature, and salinity levels [32,33].

The Ω3 fatty acid biosynthesis can be stimulated by a number of environmental stresses, such as low temperature, change of salinity, or UV radiation. For example, *Pavlova lutheri* increased its relative EPA content from 20.3 to 30.3% dw when the culture temperature was reduced to 15 °C. Similarly, *Phaeodactylum tricornutum* has a higher EPA content when the temperature is shifted from 25 °C to 10 °C for 12 h, and when stressed with UV light (up to 20% of dw) [33]. Some of the increased PUFAs are used to repair membrane damage, but as PUFAs contain many double bonds, they also act as an antioxidant by scavenging free radicals. Salinity may also regulate PUFA biosynthesis; for example, *Crypthecodinium cohnii* increases its total DHA content up to 56.9% of total fatty acids when cultured in 9 g L^−1^ NaCl [32,33].

Apart from external stresses, metabolic engineering is another promising approach to increase the production of fatty acids in microalgae. Genes encoding key enzymes involved in fatty acid biosynthesis have been identified in *Ostreococcus tauri*, *Thalassiosira pseudonana*, *Phaeodactylum tricornutum,* and *Chlamydomonas reinhardtii*.

The most common industrial method for lipid extraction from macroalgae is the mechanical press or hexane leaching, though new methods are under development to increase the lipid extraction yields in an environmentally-friendly approach. These methods include ultrasound-assisted extraction, microwave-assisted extraction, and supercritical fluid extraction; though not already feasible at an industrial scale, they are gaining new interest due to the depletion of fossil fuels, which has given rise to the demand for alternatives. Macroalgae fatty acid extraction, mainly because of their minor abundance, has very few advantages compared with microalgae [34,35].

### 2.3. Polyphenols

Phenolic compounds act mainly as antioxidant and anti-inflammatory agents. They are chemically characterized as molecules containing hydroxylated aromatic rings, having the hydroxyl group attached directly to the phenyl, substituted phenyl, or another aryl group [36]. The ecological function of phenolic compounds in microalgae has been barely investigated. Macroalgae such as *Gracilaria*, *Palmaria palmata*, *Halimeda* sp., *Ecklonia cava*, *Sargassum wightii*, and *Himanthalia elongata* have been studied [37,38,39]. Among the few phenolics already identified, there are bromophenols, flavonoids, catechins, and phlorotannins [6,40]. The bromophenols already identified in the genus *Gracilaria* are simple bromophenols with just one benzene ring [41]. Flavonoids, molecules with variable phenolic structures, were detected in *Palmaria palmata*. Flavonoids are considered indispensable molecules due to their antioxidative, anti-inflammatory, anti-mutagenic, and anti-carcinogenic properties coupled with their capacity to modulate key cellular enzyme functions. Phlorotannins are found in brown algae such as kelps and rockweeds or sargassacean species, and in a lower amount also in some red algae. Their market value is still very low due to the scarce industrial attention.

### 2.4. Polysaccharides

Polysaccharides are long-chain polymeric carbohydrates (molecules composed only of carbon, oxygen, and hydrogen) composed of monosaccharide units bound together by glycosidic linkages. Algae contain large amounts of structural polysaccharides (e.g., cellulose, hemicellulose, and lignin), storage polysaccharides (e.g., glucan, fucoidan, agar, carrageenan), and mucopolysaccharides (e.g., porphyran) that the human body and most terrestrial plants are not able to produce [10,15,16].

Most of these polysaccharides are not digested by humans and, therefore, can be defined as dietary fiber, i.e., physiologically beneficial non-digestible carbohydrates [42]. Dietary fiber in algae is classified into two types, insoluble, such as cellulose, mannans, and xylan, and water-soluble such as agars, alginic acids, furonans, laminarian, and porphyran. The proportion of these two fractions determines the properties of the fiber. Soluble fiber is characterized by its ability to form viscous gels in contact with water in the intestinal tract; it is fermented in a high proportion, and its main properties are related to the decrease of cholesterol and glucose in the blood and the development of intestinal microbiota. Insoluble fiber does not form gels in contact with water but is capable of retaining water within its structural matrix, producing an increase in fecal mass that accelerates intestinal transit with a marked laxative and intestinal regulating effect [43,44]. In algae, the proportion of dietary fiber is considerable, ranging from 36% to 60% of the dry matter, with soluble dietary fiber being very high (approximately 55–70%) compared to terrestrial vegetables. For example, in red algae such as *Chondrus* and *Porphyra,* the soluble fiber content is about 20% dw, higher than that of insoluble fiber. On the other hand, brown algae such as *Fucus* or *Saccharina* have a higher insoluble fiber content (40% dw) with respect to soluble fiber [45,46].

In unicellular Rhodophyta such as *Porphyridium* sp. and *Rhodella* sp., these polysaccharides are a constituent of the cell wall; they are highly sulfated and consist mainly of xylose, glucose, and galactose. These compounds selectively inhibit the reverse transcriptase enzyme of the human immunodeficiency virus (HIV) and its replication in vitro. Sulfated polysaccharides produced by *Porphyridium aerugineum* act as a coating material on the surface of sanitary items to prevent infection from epidemic disease, e.g., COVID-19 [47].

Among the polysaccharides, β-glucans are present inside the algae as storage compounds or wall components. These polysaccharides consist of linked glucopyranosyl units. They differ in structure, size, branching frequency, and conformation, and all these features influence their physiological functions and biological activity. The selling price for β-glucans sold as immunostimulants can be more than USD 0.5 million t^−1^ [7].

The simplest structure of β-glucans consists of a linear chain of glucopyranosyl units linked by β-(1,3)-D-glycosidic bonds; this structure is present in euglenophycee, such as *Euglena* sp., *Astasia* sp., and *Peranema* sp. and haptophytes, such as *Rebecca salina*. Another simple structure, consisting of a chain of glucopyranosyl units linked by alternate β-(1,3)- and β-(1,4)-D-glycosidic bonds, is present in chlorophyceae (e.g., *Ulva lactuca)*, xantophyceae (e.g., *Monodus subterraneum)*, rhodophyceae (e.g., *Kappaphycus alvaretzii)*, and dynophyceae (e.g., *Peridinium gatunense)*. A more complex structure consisting of a linear β-(1,3) chain with β-(1,6) side chain branches is present in phaeophyceae (e.g., *Laminaria* sp. and *Eicenia* sp.), in chrysophyceae (e.g., *Ochromonas* sp.), in raphidophyceae (e.g., *Haramonas dimorpha)*, and bacillariophyceae (e.g., *Chaethocerous mulleri* and *Pheaodactylum tricornutum)*. Linear β-(1,3)- and β-(1,3;1,4)-β-glucans are mainly insoluble compounds, while side chain branched β-(1,6;1,3)-β-glucans are mainly soluble [42].

β-glucans function as pathogen-associated molecular patterns (PAMPs) and can be non-specifically recognized by pattern recognition receptors (PRRs) present on the surface of the innate immune system cells. β-glucans have a potent immunomodulating activity, and their action is mediated through receptors such as Dectin-1 (a C-type lectin receptor), Toll-like receptors, complement receptor 3, scavenger receptor, and lactosylceramide. Dectin-1 is indicated as the preferential receptor for β-1-3-linked linear glucans. When the effector links to Dectin-1, the innate immune response is activated, which leads to the production of both ROS and inflammatory cytokines through the activation of transcription factors such as nuclear factor kappa-light-chain-enhancer of activated B cells (NF-kB), enzymes such as phospholipase C, and mitogen-activated protein kinases [48]. This ability to enhance defense mechanisms against infection and simultaneously down-regulate inflammations make β-glucans a promising alternative to the mainstream use of immunosuppressive drugs for inflammatory diseases.

**Figure 1:**
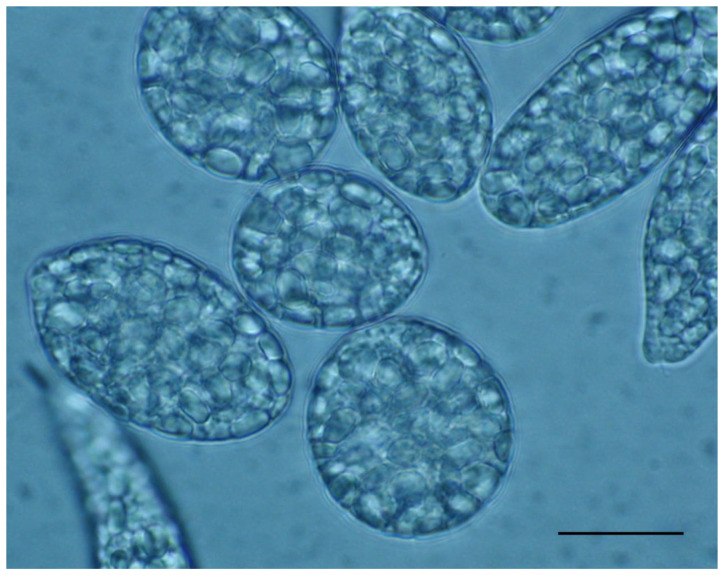
Cells of *Euglena gracilis* WZSL mutant: the very big paramylon granules fill up the cytoplasm. Scale bar: 30 μm.

Among insoluble β-glucans, the one synthetized by *Euglena* sp. and other euglenoids deserves mentioning (Figure 1) [17,43,44,49]. This linear β(1,3)-glucan, termed paramylon, is present in granular form in various locations inside the cell; in some species, the granules are scattered throughout the cytoplasm, while in others, they can be massed together. In some other species, granules are few but large and located in a fairly constant position. The shape and size of granules differ markedly and, together with their distribution inside the cell, represent a taxonomic feature. Paramylon has an unusual high crystallinity as a natural macromolecule. This high crystallinity is an advantage in that paramylon granules can be isolated from *Euglena* cells and axenically cultivated under controlled conditions in fermenters. The procedure is efficient, very low cost, and consists of simply disrupting the cells and purifying the granules by successive washing with a low concentration of detergent [17]. Paramylon crystallinity is due to higher order aggregates of nanofibrils, measuring 4–10 nm, composed of unbranched triple helices of β-(1,3)-D-glucan chains [42].

Paramylon, either in granules or as nanofibers, has been used to assess the beneficial potential of β-glucans in different experimental models involving plants, animals, and humans and investigate the structure-function relationship and mechanism of action.

In its granular form, paramylon was tested on *Artemia* shrimps upon addition to the culture medium. It proved effective in mitigating the negative effects of stressors (e.g., deteriorated environmental conditions), enhancing the survival performance of both adults and offspring, and increasing the number of offspring, hence the reproductive success of the population. The effect of the glucan may be due to the entry of paramylon granules into the digestive tract of the shrimps, which enhances the production of cell activating factors in the hemocytes, increasing the phagocytic activity of the granulocytes, thus providing defense against diseases. Paramylon could be used as a non-specific dietary additive in aquaculture feed, improving its nutritional value, mitigating the negative effects of stressors when they occur, and promoting innate immunity of the animals [50].

Paramylon nanofibers were tested in human peripheral blood mononuclear cells (PBMC) to verify the β-glucan capacity to activate the innate immune system response. Paramylon nanofibers increased transactivation of the nuclear factor k-light-chain-enhancer of activated B cells (NF-kB), which is the rapid-acting primary transcription factor, i.e., the first responder to harmful stimuli, as shown by the evident immunofluorescence nuclear labeling of treated cells. NF-kB, in turn, increases the expression of pro-inflammatory mediators (TNF-α, IL-6, COX-2, and iNOS). These mediators are highly inflammatory and indispensable when priming immune responses and licensing dendritic cells. Paramylon nanofibers also induced the production of a high level of NO and exerted an inhibitory effect on cytokine expression via inhibition of NF-kB transactivation, thus preventing a dangerous cytokine storm. This signaling cascade guarantees a safe activation of the innate immune system, as demonstrated by the presence of newly differentiated dendritic cells [48].

Paramylon nanofibers also have an anti-fibrotic effect. The administration of paramylon nanofibers (via intraperitoneal injection) in mice with liver fibrosis induced by treatment of CCl_4_ ameliorated the overall clinical picture of the animals, dampening the CCl_4_-induced loss of weight and preventing the increase of aspartate aminotransferase typical of hepatocyte damage. It also restored the normal tissue consistency and appearance of the organ; in fact, CCl_4_-treated liver had a trabecular ‘fishnet’ texture typical of mild to severe edema, while normal color and consistency were restored in paramylon-treated livers, indicating a relieved hepatic injury. Collagen deposition in the liver of paramylon-treated animals was lower than in CCl_4_-treated animals. Moreover, the nanofiber treatment greatly reduced the overall alteration of tissue parenchyma, and the lobular architecture was alike that of non-damaged liver, with mild hepatocyte ballooning with respect to CCl_4_-damaged livers and almost no necrosis regions nor infiltration of inflammatory cells. The inhibiting action of hepatic γδT cells upon Dectin-1 ligation by paramylon nanofibers, which promotes hepatocyte regeneration, reduces the inflammation and collagen production by liver stellate cells [18].

Paramylon nanofibers also proved effective in counteracting low water availability (i.e., drought) stress in tomato plants. Flowering and fruit ripening of stressed paramylon-treated plants were precocious with respect to untreated well-watered plants, whereas fruits of stressed untreated plants do not ripen beyond the green ripening stage. Thanks to paramylon action, the optimal plant water regimen could be lower by more than 10 times. Ecophysiological parameters (i.e., leaf water potential, stomatal conductance, and photosynthetic yield) were dramatically influenced by water stress, all of them undergoing a continuous decrease to saturation. Root treatment with paramylon nanofibers allowed all the parameters to recover to the values of well-watered plants. These results indicate an effective action of paramylon nanofibers on stomatal behavior, whose control improves water use efficiency, hence preventing dehydration. This action is associated with a transient modification of the content of main plant hormones, i.e., abscisic acid, jasmonic acid, and salicylic acid. The great increase of physical-chemical and quality parameters such as the antioxidant compounds (Vitamin A/C/E, lycopene, β-carotene, and phenols) together with the increase of carbohydrates (glucose, fructose, and sucrose) in the fruits of paramylon-treated plants improved their nutritional value and sensory quality. Moreover, the higher dry matter content (i.e., lower moisture) allowed a better post-harvest storage capability, extending the commercial period and increasing the commercial product value [51,52].

### 2.5. Vitamins

Vitamins are essential organic micronutrients that humans and animals cannot synthesize directly in sufficient quantities and so must obtain from the diet [13]. These compounds serve as precursors for essential enzyme cofactors necessary for metabolic functions. Examples of these essential compounds are fat-soluble pro-vitamin-A, vitamin E (α-tocopherol) (a water-soluble B-group vitamin), and vitamin C (ascorbic acid). Vitamin A plays an essential role in reproductive functions, embryonic development, growth, effective vision maintenance, and immune system functioning. Group B vitamins protect against anemia and skin problems and exert a direct effect on energy levels and brain function. Vitamin C provides protection against immune system deficiencies, cardiovascular disease, prenatal health problems, eye disease, and even skin wrinkling. Vitamin E is the major lipid-soluble component in the cell antioxidant defense system, shown to be effective against oxidation-linked possible conditions and diseases, including cancer, aging, arthritis, and cataracts [21].

In macroalgae, the highest amount of vitamin A content (expressed as amount of pro-vitamin A, i.e., β-carotene) is found in *Porphyra vietnamensis*, with about 258 mg kg^−1^ dw, followed by *Codium fragile* (198 mg kg^−1^ dw), and *Gracilaria chilensis* (114 mg kg^−1^ dw). In microalgae, the highest amount is present in *Tetraselmis suecica* (296 mg kg^−1^ dw), followed by *Dunaliella tertiolecta* (83 mg kg^−1^ dw).

Vitamins of the B-group (B_1_-B_3_, B_5_-B_7_, B_12_) are present in almost all macroalgae and microalgae. Significant amounts of B_1_ are reported in *Chondrus ocellatus* (90 mg kg^−1^ dw) and *Tetraselmis suecica* (34 mg kg^−1^ dw); vitamin B_2_ is higher in microalgae than in macroalgae, with about 30 mg kg^−1^ dw in *Isochrysis galbana* and *Dunaliella tertiolecta* and about 1 mg kg^−1^ dw in *Ulva fasciata*. The distribution is almost the same for vitamins B_5_-B_7_, while vitamin B_3_ is present in higher amounts in macroalgae, reaching 2 g kg^−1^ dw in *Caulerpa lentillifera*. As to vitamin B_12_ (cobalamin), it is present in macroalgae such as *Ulva* and *Porphyra* (0.1 g kg^−1^ dw), which is the same concentration of cobalamin present in the liver; cobalamin is abundant also in the microalgae such as the haptophyte *Pavlova* (0.01 g kg^−1^ dw) [21]. The content of vitamin C in macroalgae ranges from 3 g kg^−1^ of dw of *Enteromorpha flexuosa* to 13 g kg^−1^ dw of *Laminaria*, while in microalgae ranges from 5 g kg^−1^ dw in the chlorophyte *Nannochloris* to 18 g kg^−1^ of dw of the diatom *Chaetoceros*, with a great inter and intraspecific variability. Vitamin E in the form of α-tocopherol is present in macroalgae such as *Macrocystis pyrifera* in amounts comparable with plant oil (1.3 g kg^−1^ lipid) [6]; in microalgae, high vitamin content is found in the chlorophytes *Tetraselmis* (6.3 g kg^−1^ dw), *Chlamydomonas* (4 g kg^−1^ dw), and *Dunaliella* (2 g kg^−1^ dw) [13,21,53].

Though vitamin profiles of algae can vary according to algal species, season, growth stage, and environmental factors, algae can be considered a functional source of essential compounds to fulfill the dietary requirements of humans and animals as food or feed complements. Vitamins from algae are not purchased as single biomolecules but as part of other algal supplements (food and nutraceutical); hence, their market value is not easy to assess [7].

## 3. Conclusions and Perspective

Algae are turning into one of the most attractive natural sources for various categories of primary and secondary metabolites with a wide range of possible applications. These compounds, characterized by bio-activity, can be both health-promoting and disease-suppressing; hence, they can be used as dietary supplements in food and as therapeutic adjuvants for the management of many different diseases.

Polyunsaturated fatty acids, polysaccharides, carotenoids, phycobiliproteins, and other metabolites are only some of the many metabolites that have been tested in clinical and pharmacological studies. In the last decades, there has been a surge in demand for healthier, more natural, sustainable products to bio-fortify food, boost the immune system without the need for drugs, replace synthetic antibiotics with novel compounds already present in nature, and have the same or higher efficacy against antibiotic resistance pathogens. Algae are one of the best approaches to addressing nutritional deficiencies of many foods and feeds and a promising alternative to animal sources for vegetarian and vegan consumers. However, although numbers of algal bioactive molecules are commercially available and tested, the social acceptance of health benefits is still uncertain [54].

Macroalgae present advantages over microalgae due to the lower cost of cultivation, especially in the offshore set, which prevents the utilization of land, and do not compete with agriculture crops, and to the high amount of achievable biomass. Additional nutrients are not generally needed; the carbon footprint is negligible and limited to the biomass processing steps, as well as the risk of eutrophication of aquatic systems, making macroalgae production and environmentally-friendly exploitation. Moreover, due to their high capacity for carbon fixation, macroalgae have the potential to act as bioremediators of eutrophic coastal waters. At present, the main concern is to prevent the overexploitation of wild macroalgae stocks by reducing the environmental pressure caused by the growing demand for this natural resource. This goal can be achieved by efficient aquaculture systems providing controlled or standardized products with a nutritional profile comparable to that of the wild stock, with minimum variation along the seasons.

Microalgae present advantages over macroalgae due to the abundance of bioactive compounds already tested in many years of research and investigation. The main applications of microalgae aquaculture have always been (and still are) as feed for the growth of larvae and juvenile shellfish and finfish, to increase their nutritional value, and to improve survival and immune defenses. These effects are also increasingly exploited for human health. In the clinical daily routine, the most important expected progress in this field would be an increased use of algal polysaccharides such as alginate gels as wound healing, antiulcer agents, and the β-glucans (paramylon nanofibers) as immunostimulants. These molecules, like all other bioactive compounds of algal origin, would be best utilized when novel extraction and purification methodologies are developed to allow sustainable, cost-effective industrial utilization [15].

So far, the limitations of developing industrial algae biotechnology are mainly represented by the high downstream process and bio-refinery costs of the biomass, which can account for up to 50–80% of the whole amount. Because of these limitations, research efforts should focus on advancing farming methodologies, new biomolecules identification and characterization, high yields extraction methods, and low energy consumption processes.

## Figures and Tables

**Table 1:** Example of some bioactive compounds present in micro and macroalgae (in red). References are indicated only for the algae and corresponding bioactive molecules not cited in the text. When the species is lacking, it is because the reference does not give an indication of the species.

Category	Bioactive Molecule	Microalgae and Macroalgae
*Chlorophylls*	chlorophyll *a*	*Chlorella* sp.
*Phycobiliproteins*	allophycocyanin	*Arthrospira* sp.
	phycocyanin	*Arthrospira* sp.
	phycoerythrin	*Porphyra* sp.
	phycoerythrocyanins	*Pyropia tenera*
*Carotenoids*	astaxanthin	*Haematococcus* sp.
	β-carotene	*Dunaliella salina*
	fucoxanthin	*Phaeodactylum tricornutum* *Undaria pinnatifida* *Scytosiphon lomentaria* *Petalonia binghamiae* *Laminaria religiosa*
	siphonoxanthin	*Codium fragile*
	lutein	*Porphyra tenera*
	zeaxanthin	*Ascophyllum nodosum*
*PUFAs*	arachidonic acid (ARA)	*Parietochloris incisa* [20] *Porphyridium cruentum* [20] *Rodomella subfusca* [20] *Gracilaria* sp. [20] *Ceramium rubrum* [20]
	docosahexaeonic acid (DHA)	*Crypthecodinium cohnii* *Ostreococcus tauri* *Thalassiosira pseudonana*
	eicosapentaenoic acid (EPA)	*Phaeodactylum tricornutum* *Nannochloropsis* sp. *Dunaliella* sp. *Pavlova lutheri* *Nitzschia* sp.
*Polyphenols*	bromophenols	*Gracilaria* sp.
	flavonoids	*Palmaria palmata*
	catechins	*Halimeda* sp.
	phlorotannins	*Ecklonia cava*
*Polysaccharides*	alginic acid	*Undaria pinnatifida* [15] *Saccharina latissima* [15]
	carrageenan	*Chondrus crispus* [15] *Euchema cottoni* [15] *Gigartina skottsbergii* [15]
	fucoidan	*Macrocystis* sp. [15] *Saccharina japonica* [15] *Undaria pinnatifida* [15]
	paramylon	*Euglena gracilis* *Astasia* sp. *Peranema* sp. *Rebecca salina*
	laminarian	*Laminaria* *Fucus vesicolosus*
	porphyran	*Porphyra* sp.
	ulvan	*Ulva lactuca* *Monostroma* sp.
*Vitamins*	Vit A	*Dunaliella tertiolecta* *Tetraselmis suecica* *Gracilaria chilensis* *Porphyra vietnamensis* *Codium fragile*
	Vit B	*Dunaliella tertiolecta* *Pavlova* sp. *Tetraselmis suecica* *Isochrysis galbana* *Chondrus ocellatus* *Ulva fasciata* *Caulerpa lentillifera*
	Vit C (ascorbic acid)	*Nannochloris* sp. *Chaetoceros* sp. *Enteromorpha flexuosa* *Laminaria* sp.
	Vit E (tocopherol)	*Tetraselmis* sp. *Chlamydomonas* sp. *Macrocystis pyrifera*
	Vit K	*Anabaena cylindrica* [21] *Grateloupia turuturu* [21]

## Data Availability

Not applicable.
